# Airway management in out-of-hospital cardiac arrest in Finland: current practices and outcomes

**DOI:** 10.1186/s13049-016-0235-2

**Published:** 2016-04-12

**Authors:** Pamela Hiltunen, Helena Jäntti, Tom Silfvast, Markku Kuisma, Jouni Kurola, for the FINNRESUSCI Prehospital study group

**Affiliations:** Centre for Prehospital Emergency Care, Kuopio University Hospital, PO Box 1777, FIN-70210 Kuopio, Finland; EMS, Department of Emergency Care, Helsinki University Hospital, Stenbäckinkatu 9, 000209 HUS Helsinki, Finland

**Keywords:** Out-of-hospital cardiac arrest, Airway management, Prehospital cardiac arrest

## Abstract

**Background:**

Though airway management methods during out-of-hospital cardiac arrest (OHCA) remain controversial, no studies on the topic from Finland have examined adherence to OHCA recommendations in real life. In response, the aim of this study was to document the interventions, success rates, and adverse events in airway management processes in OHCA, as well as to analyse survival at hospital discharge and at follow-up a year later.

**Methods:**

During a 6-month study period in 2010, data regarding all patients with OHCA and attempted resuscitation in southern and eastern Finland were prospectively collected. Emergency medical services (EMS) documented the airway techniques used and all adverse events related to the process. Study endpoints included the frequency of different techniques used, their success rates, methods used to verify the correct placement of the endotracheal tube, overall adverse events, and survival at hospital discharge and at follow-up a year later.

**Results:**

A total of 614 patients were included in the study. The incidence of EMS-attempted resuscitation was determined to be 51/100,000 inhabitants per year. The final airway technique was endotracheal intubation (ETI) in 413 patients (67.3 %) and supraglottic airway device (SAD) in 188 patients (30.2 %). The overall success rate of ETI was 92.5 %, whereas that of SAD was 85.0 %. Adverse events were reported in 167 of the patients (27.2 %). Having a prehospital EMS physician on the scene (*p* < .001, OR 5.05, 95 % CI 2.94–8.68), having a primary shockable rhythm (*p* < .001, OR 5.23, 95 % CI 3.05–8.98), and being male (*p* = .049, OR 1.80, 95 % CI 1.00–3.22) were predictors for survival at hospital discharge.

**Conclusions:**

This study showed acceptable ETI and SAD success rates among Finnish patients with OHCA. Adverse events related to airway management were observed in more than 25 % of patients, and overall survival was 17.8 % at hospital discharge and 14.0 % after 1 year.

## Background

In Finland, emergency medical services (EMS) attempt resuscitation in 51 out-of-hospital cardiac arrests (OHCA) per 100,000 inhabitants each year [1]. Among these patients, airway management is controversial, however, and evidence of its role related to its outcome remains poorly documented [2, 3]. Nevertheless, securing the airway for sufficient oxygenation and ventilation during resuscitation is a universally accepted principle [4].

For decades, endotracheal intubation (ETI) has been considered the gold standard for advanced airway management [5]. However, ETI might not be the best technique in cases of OHCA [6, 7], for it is a highly technical skill that providers should practise regularly [8, 9]. In fact, in inexperienced hands, ETI can have life-threatening consequences [10–12], and a prolonged procedure can even interrupt effective chest compressions during cardiac arrest [13].

For airway management in patients with OHCA, supraglottic airway devices (SAD) have increasingly gained popularity as first-choice devices [14, 15]. Not only are they feasible for use [16, 17], but their placement also seems relatively easy and can enable chest compressions with minimal interruptions during the procedure. Successful insertion rates of SAD have been high among EMS personnel [18], and as with ETI, SAD might be associated with potential adverse events related to insertion, insertion time, and ventilation management in OHCA [19]. Furthermore, the use of advanced airway devices has been shown to cause an increased no-flow ratio during cardiac arrest [20].

In cases of OHCA, the optimal airway technique depends on the skills and experience of EMS personnel [21]. Though guidelines for managing the airway in the prehospital environment have been published [22] and though both ETI and SADs are used for cardiac arrest patients in Finland, no studies on the topic have examined the adherence to those recommendations in actual practice. Information regarding which airway devices EMS personnel use during OHCA and the extent of subsequent adverse events thus remains unknown.

In response, the primary aim of this study was to characterise the choice of available interventions for OHCA, their success rates, the verification methods used to confirm the right place of endotracheal tubes, and adverse events in the airway management process. The secondary aim was then to study patients’ survival at hospital discharge and after 1 year.

## Methods

### Study design

This prospective observational cohort study was conducted with all OHCA patients in southern and eastern Finland from 1 March to 31 August 2010. The Institutional Review Board of Helsinki University Hospital approved the study protocol (80/13/03/02/09, ClinicalTrials.gov ID NCT01295424).

### Study area and population

**Fig 1:**
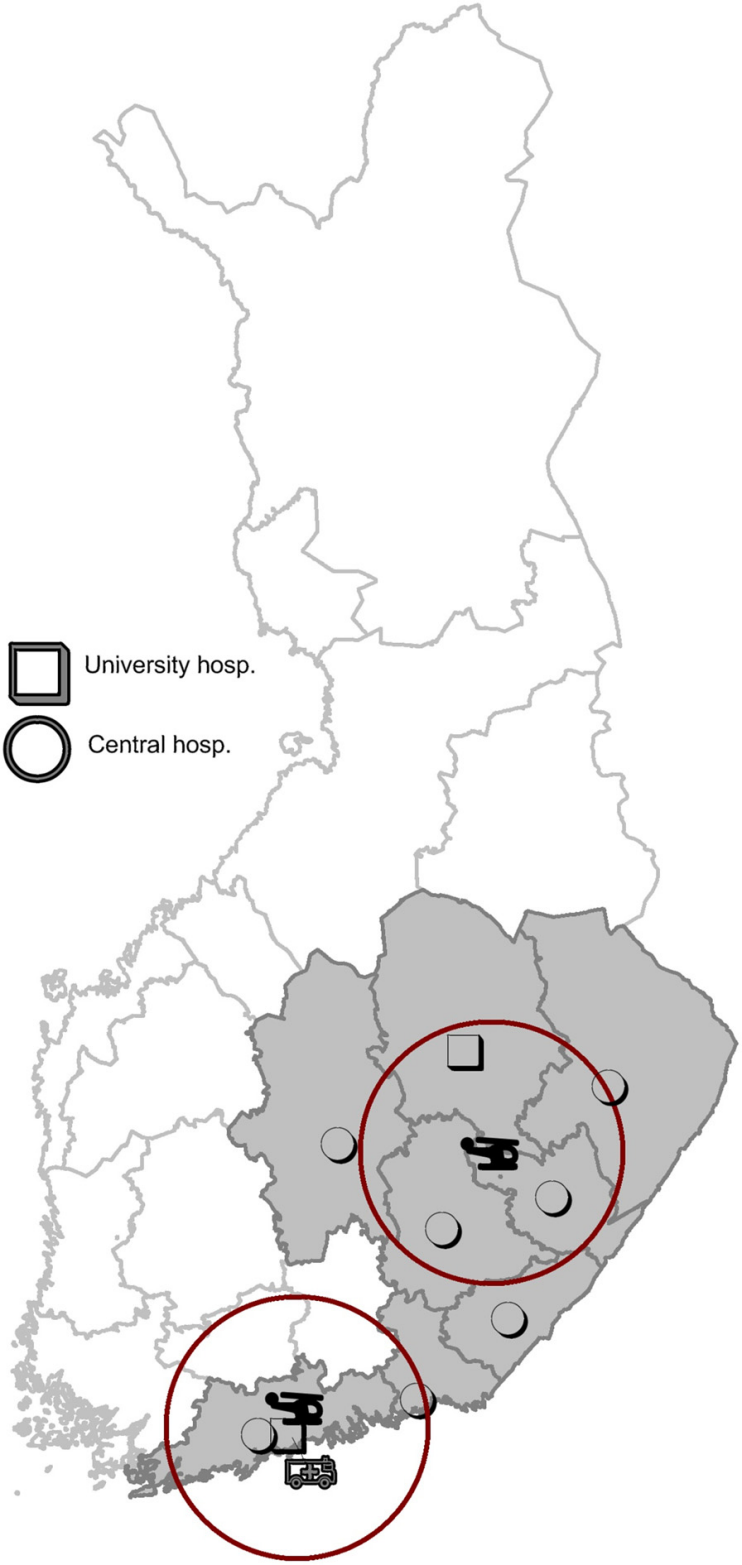
FINNRESUSCI study area

With an area of 337,000 km^2^ and population density of only 17/km^2^, Finland is a sparsely populated country in the northeast corner of the European Union. The study area encompassed a population of 2,644,200, or 49.1 % of the country’s total population, concentrated in southern Finland, where close to 600,000 people live in Helsinki, Finland’s largest city and capital. The study area also included two university and six central hospitals (Fig. 1).

### Finnish EMS system

Organised among 20 hospital districts, the three-tiered Finnish EMS system involves basic and advanced life support units (BLS and ALS), five regional physician-staffed emergency helicopter units that are always on-call, and an EMS physician-staffed ground unit in Helsinki (Fig. 1). All physicians, either specialists in anaesthesiology and intensive care or in the final stage of specialisation, are dispatched along with other EMS units to all high-risk trauma and non-trauma 112 calls. At the time of data collection, two physician-staffed helicopter units and the ground unit operated in the study area; helicopter units were dispatched to all OHCAs within 30 min, yet might be unavailable for each cardiac arrest due to weather restrictions and other tasks. The third tier—that of physicians—might not become involved in an OHCA task based on information communicated by the first or second tier at the patient’s side. At times, not needing a physician is due to the futility of the situation, often due to extensive time from collapse to EMS arrival, unsuccessful resuscitation efforts, and the presence of comorbidities.

Each of the 20 hospital districts in Finland has an administrative EMS medical director. Although national recommendations for prehospital care in OHCA are available [23–25], their local implementation is dictated by these EMS directors and thus varies. The airway technique used in OHCA depends on the education and training of the EMS provider (Table 1), as well as on the orders issued by medical directors. For advanced EMS personnel, there could be multiple airway techniques from which to choose.

**Table 1:** Description of EMS in Finland and airway devices used for OHCA patients

Tier	Staff	Background/education	Airway technique in OHCA
First tier	• First responders • Basic-EMT	• Formal training for SAD use, not necessarily with healthcare educational background • Firefighter-EMT • Practical nurse in prehosital emergency care	• SAD in eastern part of Finland • SAD/ETI in southern part of Finland
Second tier	• Advanced level	• Registered nurse (bachelor) in emergency care	• ETI/SAD • ETI/SAD
Third tier	• EMS physicians	• >90 % anaesthesiologists or residents in anaesthesiology	• ETI

*OHCA* out-of-hospital cardiac arrest, *SAD* supraglottic airway device, *ETI* endotracheal intubation, *EMS* emergency medical service, *EMT* emergency medical technicians

In eight regional dispatch centres inside the study area, trained dispatchers answer emergency calls. If a patient is not awake or not breathing normally, then dispatchers process the call as a cardiac arrest. All dispatchers at these centres are trained to give telephone-guided cardiopulmonary resuscitation (CPR) instructions.

### Data collection

This study constituted a part of the observational FINNRESUSCI study [1]. All cardiac arrest patients who underwent attempted resuscitation during a 6-month data collection period were included, regardless of age or the aetiology of the cardiac arrest. We considered resuscitation to have been attempted unless the EMS crew immediately discontinued basic CPR after its initial assessment, namely due to the futility of the situation. Further analyses were conducted on patients whose airways were managed with bag–valve–mask ventilation (BVM), SAD, or ETI. The EMS personnel documented the first airway device chosen, whether it was later replaced, and the education of the provider who ultimately managed the airway. Also reported were the highest-level EMS provider on the scene, adverse events while securing the airway, and the method of verifying the correct placement of the ET tube. During data collection, neither capnometry nor capnography was mandatory in all EMS units.

For ETI, traditional laryngoscopy was performed, and various supraglottic airway devices were used by EMS. Laryngeal tubes (VBM Medizintechnik®) were primarily used in the eastern part of the study area, whereas laryngeal masks or i-Gels® were more commonly used in the southern part. Due to the estimated small sample size of the study, all SADs were deemed supraglottic devices.

Adverse events associated with airway management were defined in the study protocol as more than two attempts, vomiting or regurgitation, or other. Events in the last category were reported as overall adverse events unrelated to airway technique selected, and multiple answers were allowed. Survival was reported in terms of which prehospital airway technique was ultimately used (e.g., ETI or SAD).

For the purposes of the study, a standardised case report form was distributed to all EMS providers. The forms were returned by fax or mail to a research nurse who entered the data into the electronic FINNRESUSCI database. Information regarding patients’ status at the time of hospital discharge was provided by the National Institute for Health and Welfare, whereas data regarding survival status at 1 year were provided by the Finnish Population Information System. Pittsburgh Cerebral Performance Category (CPC) status at 6–12 months was reported for resuscitated patients admitted to the hospital and to the intensive care unit (ICU) by a neurologist. We were unable to follow-up on the neurological outcome of patients admitted to any non-ICU ward.

### Data analysis

Data were analysed using the Statistical Package for the Social Sciences version 19.0 (SPSS Inc., Chicago, Illinois, USA) and are here presented either as medians with interquartile range (IQR) or as frequencies and percentages. The association between categorical variables was evaluated using cross tabulation and chi-square testing, and analysis of variance was used when variables were continuous. In all cases, a *p* value less than 0.05 was considered to be significant. We compared patients’ demographic and cardiac arrest characteristics according to ultimate airway technique (e.g., ETI or SAD). Logistic regression was used to identify factors related to survival at hospital discharge and at 1-year follow-up; those factors included gender, initial primary rhythm, location of cardiac arrest (i.e., at home or in public), witnesses (i.e., bystanders or EMS), whether the even was witnessed by EMS, presumed cardiac aetiology, whether the dispatcher recognised OHCA, whether CPR was provided by any bystanders before EMS arrival, the highest level of the EMS provider on the scene, ETI or SAD as the ultimate airway technique, type of region, and presence of a prehospital EMS physician in OHCA patient management.

## Results

### Demographic data

**Fig 2:**
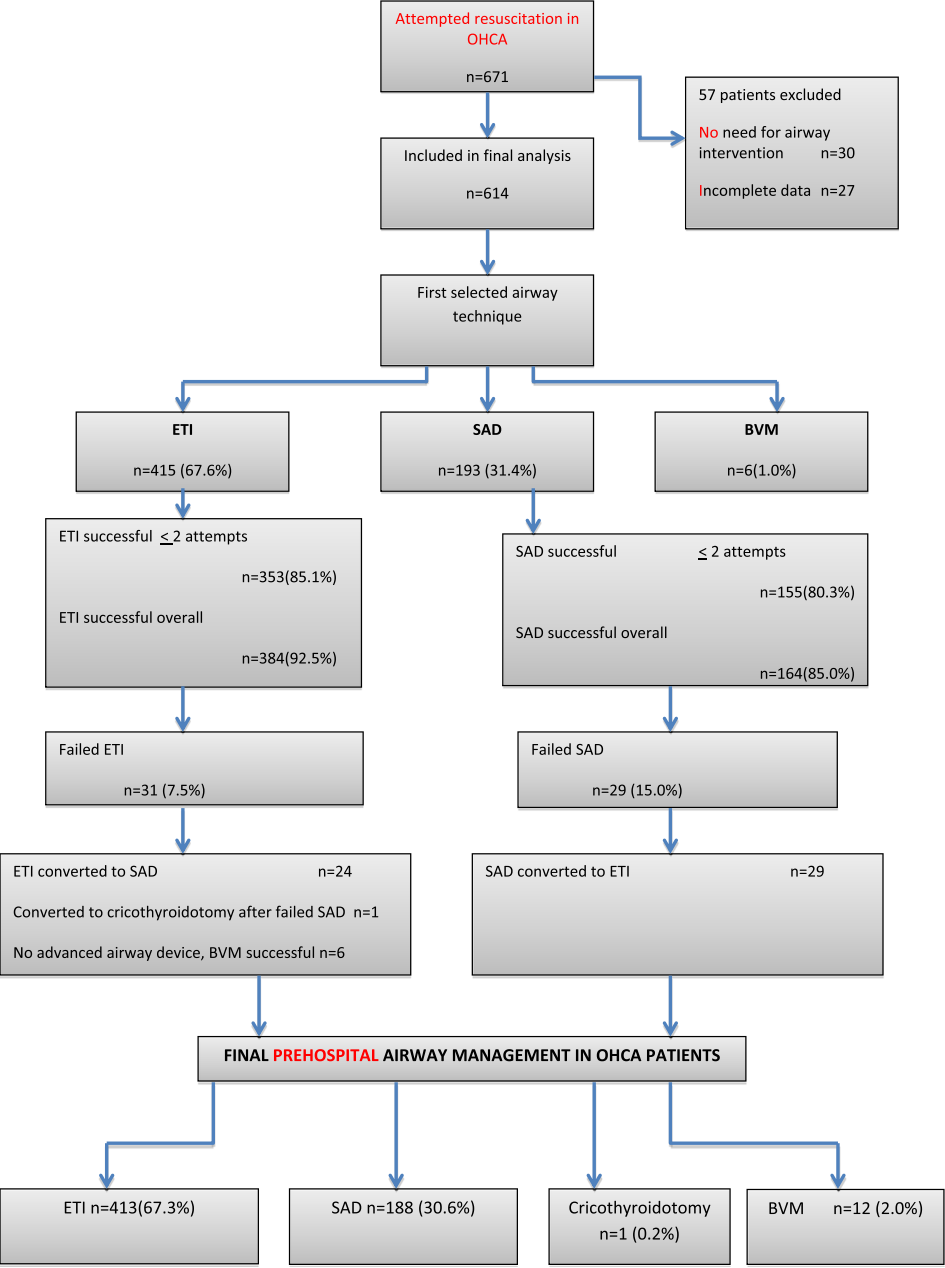
Study-flow-chart

During the 6-month study period, Finnish EMS crews attempted resuscitation in 671 OHCA patients, for an incidence of 51 attempted resuscitations per 100,000 inhabitants per year. Airway management data of 27 patients were missing, and 30 patients who were conscious and breathing upon EMS arrival did not need any airway intervention. In all, 614 patients were included in the final analysis (Fig. 2).

The median age of patients was 66 (IQR 56–78) years, and 435 of them were male (70.8 %). The cause of arrest was considered to be of cardiac origin in 324 patients (52.8 %), and the primary rhythm was shockable in 183 patients (29.8 %). OHCA was witnessed in 560 patients (91.2 %) and by EMS in 117 of them (19.1 %). In 126 patients (20.5 %), cardiac arrest was witnessed with primary shockable rhythm, though not by EMS, and was considered to be of cardiac origin. Excluding EMS-witnessed OHCAs, T-CPR instructions were provided for 152 patients (30.6 %), and 298 patients (48.5 %) received bystander CPR before EMS arrived. The characteristics of the study group appear in Table 2.

**Table 2:** Characteristics of OHCA patients and final advanced airway technique^a^

Characteristics	Total *n* = 614	SAD *n* = 188	ETI *n* = 413	*P*-value
Age, median (IQR)	66(56–78)	68(57–78)	65(55–77)	0.135
Sex, n (%) of males	436(71.0)	137(72.9)	289(70.0)	0.469
Witnessed, n (%)	560(91.2)	190(90.4)	378(91.5)	0.659
EMS-witnessed, n (%)	119(19.1)	31(16.5)	82(19.9)	0.328
CPR before EMS arrival, n (%)	298(48.5)	95(50.5)	199(48.2)	0.593
Highest EMS provider level on scene, n (%)				<0.001
Basic level paramedic	119(19.4)	85(45.2)	31(7.5)	
Advanced level paramedic	246(40.1)	78(41.5)	162(39.2)	
	249(40.6)	25(13.3)	220(53.3)	
Prehospital physician				
Shockable initial rhythm^b^, n (%)	183(29.8)	44(23.4)	136(32.9)	0.035

*SAD* supraglottic airway device

*ETI* endotracheal intubation

*EMS* emergency medical service

*CPR* cardiopulmonary resuscitation

*P*-values from chi-square test except age that was counted with one-way-ANOVA

^a^ BVM (*n* = 12) and cricothyroidotomy (*n* = 1) listed only in “total”

^b^ in two patients, the initial rhythm was not monitored

### Airway technique

The first selected airway device was ETI in 67.6 % (*n* = 415) patients and SAD in 31.4 % patients (*n* = 193). Six patients were treated with BVM without any attempt to secure the airway (1.0 %). The final prehospital airway technique was ETI in 67.3 % of patients (*n* = 413) and SAD in 30.2 % (*n* = 188). One patient had a successful cricothyrotomy after failed ETI and SAD attempts, whereas in another six patients, attempts to secure the airway failed, though BVM was succeeding in supporting oxygenation and ventilation.

Most patients (*n* = 554, 90.2 %) were treated with the initially selected airway technique only (ETI, SAD, or BVM). In 59 patients (9.6 %), two airway techniques were needed, and one patient required all three (0.2 %). Of the 614 patients, 602 (98.0 %) underwent the successful insertion of an advanced airway device, either ETI or SAD.

### Airway success rate with first selected airway device

The overall success rate of ETI was 384/415 (92.5 %) and of SAD164/193 (85.0 %), with no more than two ETI placements in 353 of all ETI patients (85.1 %) and in 155 of SAD patients (80.3 %). In patients who ultimately received ETI, the provider was of BLS level for 82 patients (19.9 %), of ALS level for 264 patients (62.4 %), and a prehospital EMS physician for 67 patients (16.2 %). In the SAD group, the corresponding figures were 129 patients (68.7 %), 57 patients (30.3 %), and two patients (1.1 %).

### Reported adverse events

EMS crews confronted adverse events while securing the airway in 167 patients (27.2 %). More than two attempts were necessary in 50 patients (8.1 %), and 44 patients regurgitated (7.2 %); in either case, the event was registered regardless of whether it happened before or after the EMS crew arrived. In 29 patients (4.7 %), EMS crews reported difficult anatomy or impaired vision as the reason for difficult airway management. Other reasons were reported in 90 patients (14.7 %), most of whom had blood or another secretion in the upper airway. EMS physicians successfully intubated eight patients after failed attempts by paramedics.

Methods of verifying the correct placement of the ET tube—multiple answers were allowed—were capnometry or capnography (*n* = 315, 76.3 %), auscultation and observing chest movements (*n* = 281, 68.0 %), and placing the tube under visual control (*n* = 356, 86.2 %). Four ETI patients had no record of how tube placement was verified.

### Prehospital EMS physicians on the scene

A prehospital EMS physician was involved in the patient management of 249 patients (40.6 %), 220 (53.3 %) of whom were ETI patients and 25 of whom (13.3 %) were SAD patients. No airway intervention was performed for four of these patients.

### Patient outcome

Of 614 patients, 213 survived event (34.7 %). Of survivors, 109 (17.8 %) survived to hospital discharge, whereas 86 (14.0 %) were alive at the 1-year follow-up. CPC at 6–12 months was available for 71 patients (65.1 %), 46 of whom had CPC 1 or 2 (42.2 % of all patients discharged). In multivariate analysis, initial shockable rhythm (*p* < .001, OR 6.96, 95 % CI 3.61–13.44) and prehospital EMS physician presence on the scene when treating the OHCA patient (*p* = .013, OR 2.57, CI 1.22–5.43) were related to survival at 1 year. The same factors were related to survival at hospital discharge: primary shockable rhythm (*p* < .001, OR 5.23, 95 % CI 3.05–8.98), prehospital EMS physician presence (*p* < .001, OR 5.05, 95 % CI 2.94–8.68.), and maleness (*p* = .049, OR 1.80, 95 % 1.00–3.22).

## Discussion

Among the chief findings of this study, the first is that EMS personnel in Finland most often treat airways in OHCAs with ETI (67.3 %) and at an acceptable overall success rate (92.5 %), as 85.1 % were successful with two or fewer attempts. Second, SAD was used as the final airway device in 30.6 % of the patients; 85.0 % of those SADs were successfully placed. Third, adverse events related to airway management were observed in approximately a quarter of all OHCA patients. Lastly, patients who survived to hospital discharge and at 1 year more likely suffered an OHCA with initial shockable rhythm and had a prehospital EMS physician on the scene during their treatment.

In agreement with a retrospective OHCA study from the United States [26], which showed that ETI was the technique most commonly used (52.6 %), we found that ETI was the most frequently used device in OHCAs in Finland. At the same time, studies from Japan have demonstrated that only a minority of OHCA patients there have been intubated [2, 3]. It thus seems that there are variations in national practices and guidelines regarding the use of different airway devices. Our study supports current hospital district recommendations, according to which all EMS providers should be trained to use some airway device in responding to OHCA and ETI should be performed primarily by ALS or prehospital EMS physicians.

In a study by Wang et al. [27], overall ETI success rates were 77.0 %, compared to a rate of 92.5 % in this study. However, their data also included non-arrest medical and trauma patients, most of whom (78.0 %) were still OHCA patients. Meanwhile, Diggs [28] reported an overall ETI success rate in cardiac arrest patients of 85.5 %. Yet, even if success rates are acceptable, EMS personnel are nevertheless liable to encounter significant challenges [29].

We found that placing the ETI seemed to be effective and lead to desirable results with acceptable rates; however, a previous survey conducted in Finland reported low frequencies of advanced airway procedures, including tracheal intubation, by non-physicians [30]. Nordic guidelines recommend that prehospital ETI should be performed in non-arrest patients only by anaesthesiologists skilled in drug-assisted ETI and that experienced ALS-trained EMS personnel may attempt the procedure during cardiac arrest, yet avoid repeated attempts [22]. In the present study, ETI was most often performed by ALS (62.4 %) and succeeded in 85.1 % of patients in two or fewer attempts.

Of the 614 OHCA patients in this study, 30.6 % had an SAD as the ultimate airway technique. In recent years, alternative airway techniques have been increasingly used as part of a primary approach for managing airways in OHCA, especially when EMS personnel are at the BLS level [31]. Despite these devices’ popularity, reports have shown that adverse events related to SAD use in OHCA are possible. For instance, a Norwegian study [19] reported high overall laryngeal tube insertion rates in OHCA (85.3 %) performed by non-physicians, yet also a great deal of insertion-related problems (52.7 %). At the same time, Länkimäki et al. [32] reported that first responders with only brief training achieved 71.9 % laryngeal tube success rates in OHCA on the first attempt, with difficulties in 14.1 % of the cases.

In this study, the overall success rate for SAD placement was 85.0 %, most of which were placed by EMS providers at the BLS level (68.7 %). This finding may suggest a need for additional training in airway management skills for BLS-level providers. Nevertheless, we believe that SAD is an effective for BLS-level providers, who would otherwise know only the BVM method, which is often a challenging manoeuvre to perform [17].

Most patients in our study needed only one airway technique (ETI or SAD, 89.3 %), and only one cricothyrotomy was necessary after failed ETI and SAD attempts. In more than a quarter (27.2 %) of OHCA patients, adverse events occurred while securing the airway—most frequently, the need for two attempts (8.1 %). This rate could be acceptable, though multiple intubation attempts have been associated with adverse events in emergency situations [10, 33].

High rates of unrecognised ETI have been reported among paramedics [34], though the problem is probably more frequent than assumed, especially when capnometry is not used, and leads to a higher mortality rate [35]. CPR guidelines consider end-tidal CO_2_ monitoring to be a compulsory method after ETI placement [22, 36] in order to prevent catastrophic events from occurring. We found that correct ETI placement was verified by capnometry in 76.3 % of intubated patients, though we did not record whether the absence of it stemmed from a lack of equipment or a lack of use. At the time of our study, Finnish guidelines advised using *capnometry* after placing an ETI [23]. For SADs and especially for BLS-level personnel using supraglottic airway devices, guidelines and practices varied among hospital districts, and it was possible that capnometry and capnography did not exist in the supplies of each EMS unit. Currently, it is *capnography* that is considered mandatory in EMS units and should be used when securing an airway either with an SAD or by ETI [25].

Overall survival from OHCA regardless of aetiology was 17.8 % at hospital discharge and, after 1 year, 14.0 %. These survival rates are consistent with those of previous reports [37]. Regression analysis showed that initial shockable rhythm and the presence of a prehospital EMS physician were factors related to survival at hospital discharge and at 1 year; however, we found a rather modest rate of prehospital EMS physician presence in some phases of care of OHCA patients (40.7 %). Physician-staffed units are routinely dispatched in primary response in cases of cardiac arrest, yet due to long distances, weather restrictions, and the limited number of these units, they cannot respond all OHCA calls.

Limited evidence suggests that the presence of a physician improves outcomes in patients with OHCAs [38]. However, to date, no randomised controlled studies on the topic exist or have been conducted on improved outcomes with any elements of advanced cardiac life support. For example, a recent study from Norway showed no difference in outcome between physician- and paramedic-staffed ambulances [39].

Regression analysis showed no relationship between the airway technique used and survival. A recent meta-analysis suggested that non-traumatic OHCA patients whose airways were treated with ETI had better outcomes than those treated with SADs [40]. Patient survival might not be an appropriate indicator for comparing different airway techniques used during cardiac arrest, however, since the outcome could be influenced by many factors [41].

### Limitations

This study poses some limitations. For one, data collection was challenging, since this study was an observational cohort study of cardiac arrest patients in the prehospital environment. We did not specify whether adverse events in airway device placement were related to ETI or SAD or whether placement succeeded on the first attempt. We recorded the final airway placement provider not as a person but as the person’s having BLS-, ALS-, or physician-level expertise, which prevented us from reporting whether the provider changed during the airway placement procedure. We also did not specify the type of SAD used, though some SAD devices can be more feasible than others. However, due to the low number of data, we decided not the split the study group by SAD, which could have added bias. Moreover, since multiple attempts should be avoided [22] and a maximum of two attempts is acceptable for airway device placement, we decided to report the result of two attempts in the study group.

Despite those limitations, our study offers a valid description of the prehospital airway management process in Finnish OHCA patients in a real-life setting.

## Conclusions

This study showed that ETI was the most frequently used airway technique in OHCA in Finland and exhibited an acceptable rate of success, which suggests that it is a feasible method for experienced EMS personnel for OHCA patients. SAD success rates were less than those for ETI. Since BLS-level providers mostly used SADs, that result suggests a need to improve their skills with training. EMS personnel encountered adverse events in airway management in every fourth patient. The study suggested that primary shockable rhythm and a prehospital EMS physician’s involvement in OHCA patient care improves survival rates, though the latter finding should be interpreted with caution.
